# Seismic detection of strong ground motions by *M*_*W*_5.6 North Korean nuclear explosion

**DOI:** 10.1038/s41598-019-41627-x

**Published:** 2019-03-26

**Authors:** Tae-Kyung Hong, Junhyung Lee, Seongjun Park, Hyun Ho Yoon, Woohan Kim, Jin Soo Shin

**Affiliations:** 1 Yonsei University, Department of Earth System Sciences, 50 Yonsei-ro, Seodaemun-gu, Seoul 120-749 South Korea; 2 Gyeongsang National University, Department of Earth and Environmental Sciences and RINS, Jinju, Gyeongsangnam-do 660-701 South Korea; 3 Earthquake Research Center, Korea Institute of Geoscience and Mineral Resources, 92 Gwahang-no, Yuseong-gu, Daejeon, 305-350 South Korea

## Abstract

The North Korean nuclear explosion test site in Punggye-ri is located in a seismically quiescent region on a stable Precambrian basement. The 3 September 2017 *M*_*W*_5.6 North Korean underground nuclear explosion (UNE) test produced unprecedented strong ground motions. The peak ground accelerations might reach tens to hundreds m/s^2^ on the surface of the UNE test site, decaying exponentially with distance. Ten shallow events with magnitudes greater than or equal to *M*_*L*_2.5 and source depths less than 3 km followed the 2017 UNE for 5 months in an area with a radius of 15 km from the UNE where strong ground shaking was experienced. The largest event with *M*_*W*_3.7 occurred 20 days after the 2017 UNE test at shallow depths less than 3 km. Its moment tensor solution indicates a combined source behavior with comparable strengths of double-couple and compensated linear vector dipole (CLVD) components, suggesting an unusual event different from typical natural earthquakes in the Korean Peninsula. The clustered shallow seismic events appeared to have occurred in damaged media that were effectively perturbed by the strong ground motions of the UNE.

## Introduction

North Korea has conducted six underground nuclear explosion (UNE) tests of increasing detonation strengths since 2000. Recent two UNE (5th, 6th) tests were made in 9 September 2016 and 3 September 2017. The 6th North Korean UNE test was the largest, producing unprecedented strong ground motions around the UNE test site. The effects of strong ground motions on regional media are crucial to assessing potential hazards. Surface subsidence and landslides at the UNE test site could possibly incorporate radioactive contamination and are therefore cause for great concern.

The UNE test site is located in a seismically quiescent region in the Korean Peninsula. The influence of successive UNE tests and strong ground motions on regional seismicity remains unclear. Further, the level of dynamic stress changes associated with the strong ground motions may trigger volcanic eruptions in Mount Baekdu, an active volcano located ~120 km away from the UNE test site. A study of the potential hazards of strong ground motions on the induction of volcanic eruptions in Mount Baekdu showed that seismic waves from a large UNE could dynamically change the internal pressure (stress) in the magma chamber, which would in turn accelerate a volcanic eruption^1^.

The geological provinces in the Korean Peninsula consists of three Precambrian massifs (Nangrim, Kyonggi, and Yongnam) and two intervening belts (Imjingang and Okchon)^2^. The Punggye-ri UNE test site is located in the northeastern Korean Peninsula, which belongs to Nangrim massif, a geologically stable region with low seismicity^3^ and a basement of high-grade gneisses and schists^2,4^.

**Figure 1:**
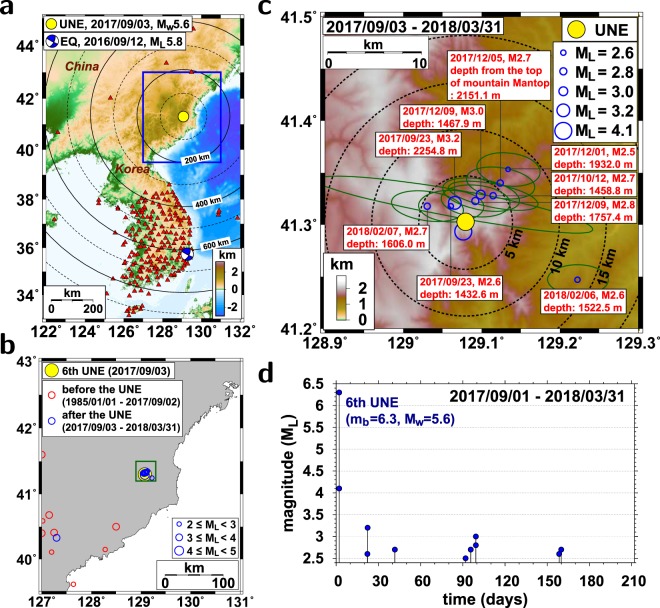
The 3 September 2017 *M*_*W*_ 5.6 underground nuclear explosion (UNE) and induced seismicity. (**a**) Map of the 2017 UNE (closed circle) and stations (triangles). The epicenter and focal mechanism solution of the 12 September 2016 *M*_*L*_ 5.8 Gyeongju earthquake are presented on the map. (**b**) Seismicity before and after the 2017 UNE. (**c**) Spatial distribution of post-UNE seismic events. The vertical source locations below the surface are annotated. The error ellipses of the epicentral locations are presented. (**d**) Time history of post-UNE seismic event occurrence since the 2017 UNE. The figure was created using GMT 4.5.14 (https://www.soest.hawaii.edu/gmt/) and Adobe Illustrator CS6 (http://www.adobe.com/kr/products/illustrator.html).

The UNE test site is located near Mount Mantap, which has a summit elevation of 2208 m^5,6^. (Fig. 1). The seismic magnitudes of the 6 UNEs were *m*_*b*_ 4.3, 4.7, 5.1, 5.1, 5.3, and 6.3 (see Supplementary Materials)^7–12^. The 6th UNE test was conducted beneath the top of the mountain, and the other five UNE tests were conducted on the southern or eastern flanks of the mountain. The UNE tests were conducted in two tunnels whose entrances were located at elevations of ~1400 m to the south of the test site^5^. According to government reports, two additional tunnels were under construction at the test site.

The influence of strong ground shaking on the induction of seismic events has been rarely examined. The North Korean UNEs were well monitored by local and regional seismic stations, providing us a unique opportunity to investigate the effects of UNE tests. We assess the strong ground motions by the UNEs, and investigate the influence on the seismicity induction.

We collected 8478 seismic waveforms from 277 stations for the 5th and 6th UNEs and post-UNE events from regional seismic stations in Korea and China and analyzed 357 waveforms from 30 near-regional stations for hypocentral-parameter inversions at distances of 185.1–530.1 km. A total of 8478 seismic waveforms were collected from 277 stations.

We additionally analyze the 12 September 2016 *M*_*L*_5.8 Gyeongju earthquake to compare the ground motions of the 6th UNE (Fig. 1). The earthquake was the largest event in the Korean Peninsula since 1978. The earthquake ruptured a fault plane of ~26 km^2^ at depths of 11–16 km^13,14^. We collect the seismic waveforms of the earthquake from 160 local and regional stations in the Korean Peninsula^13^.

## Results

### Nuclear explosion tests

**Figure 2:**
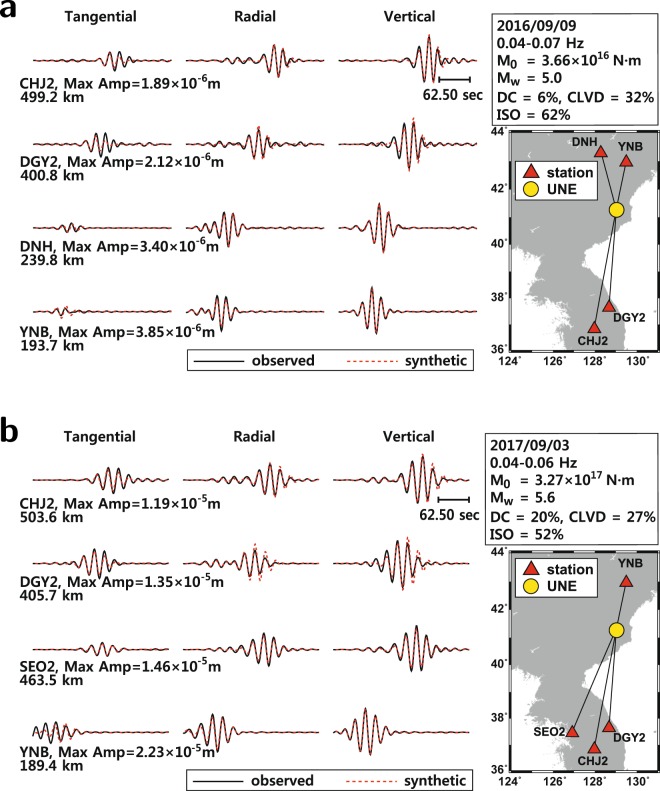
Long-period waveform inversion of North Korean underground nuclear explosions (UNEs): (**a**) the 5th UNE in 9 September 2016, and (**b**) the 6th UNE in 3 September 2017. The seismic moment of the 5th UNE is be 3.66 × 10^16^ N·m, and the moment magnitude is *M*_*W*_ 5.0. The seismic moment of the 6th UNE is 3.27 × 10^17^ N·m, and the moment magnitude is *M*_*W*_ 5.6. The figure was created using GMT 4.5.14 (https://www.soest.hawaii.edu/gmt/) and Adobe Illustrator CS6 (http://www.adobe.com/kr/products/illustrator.html).

A long-period waveform inversion is conducted to infer the moment tensor solutions of the last two (5th, 6th) UNEs (Fig. 2). The seismic moment of the 5th nuclear explosion is 3.66 × 10^16^ N · m, the magnitude is *M*_*W*_ 5.0, and the variance reduction is 91%. The energy is composed of 62% isotropic component, 32% compensated linear vector dipole (CLVD) component, and 6% double-couple component.

The seismic moment of the 6th nuclear explosion is 3.27 × 10^17^ N · m, the magnitude is *M*_*W*_ 5.6, and the variance reduction is 89% (Fig. 2). The seismic energy from the UNE is composed of 53% isotropic component, 20% double-couple component and 27% CLVD component. The 6th UNE in 3 September 2017 was stronger than the 5th UNE in 9 September 2016 by 0.6 magnitude (*M*_*W*_) units. Note that the seismic moment of the 12 September 2016 *M*_*L*_5.8 mid-crustal earthquake is 1.66 × 10^17^ N · m, and the magnitude is *M*_*W*_ 5.4^13^.

The moment tensor solutions of the UNEs suggest that they radiated azimuthally anisotropic energy, building considerable shear waves^7,15–17^. The moment magnitudes of the 6th UNE and the 12 September 2016 mid-crustal earthquake are similar, which allows us to compare the spatial distributions of strong ground motions between UNEs and natural earthquakes.

The hypocentral parameters of the 6th UNE and postseismicity are inverted using VELHYPO (see Methods). The method allows us to determine the hypocentral parameters in regions with poorly-known velocity structures^18,19^. The average *P* and S traveltime residuals are 6.1 × 10^−6^ s and 4.9 × 10^−3^ s, and their standard deviations are 0.0489 s and 0.1948 s, respectively (see Supplementary Materials). A detonation depth law suggests that the detonation depths of M5-level UNEs should be greater than ~600 m below the surface^20^. The inverted vertical source location of the 6th UNE is 597 m above the sea level, which corresponds to 1611 m below the surface considering the topography of the source region^21^. The inverted detonation depth of the 6th UNE is close to the elevation of tunnel entrance (~1400 m above the sea level and ~760 m below the surface), supporting the depth accuracy of the hypocenter-inversion method^10^.

### Strong ground motions by UNE

Natural earthquakes may accommodate fault ruptures that induce near-field effects with permanent displacements in the source regions. However, far-field ground motions are generally dominated by transient seismic waves. We can infer the levels of ground motions by seismic waves in local and regional distances. We measured the peak ground accelerations (PGAs) as a function of distance^1,22^ (see Supplementary Materials). The PGA decay rate is low in the Korean Peninsula compared to other tectonic regions^22^. We determine the PGA attenuation curve by calibrating the level of the reference PGA curve for the observed PGAs in regional distances. The observed PGA values agree with the reference PGA attenuation curve. We also determine the reference peak ground velocity (PGV) attenuation curve.

**Figure 3:**
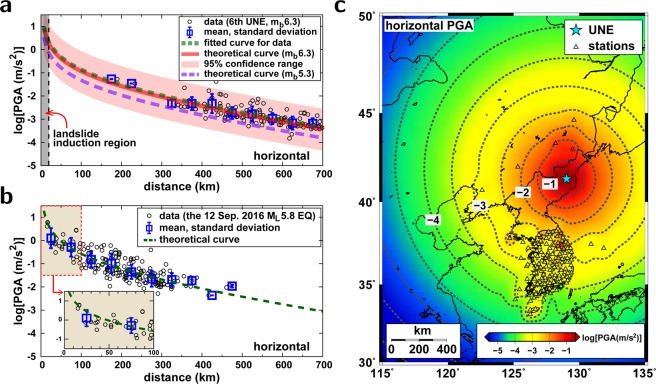
Strong ground motions induced by the 6th UNE in 3 September 2017. (**a**) Attenuation of horizontal peak ground accelerations (PGAs) of the 6th UNE as a function of distance. The observed PGAs were fitted by a curve that agrees with the theoretical reference curve for *m*_*b*_ 6.3 well. The 95% confidence range of the PGAs is marked. The horizontal PGA curve for the 5th UNE in 9 September 2016 is presented for comparison. The landslide induction regime is indicated. (**b**) Attenuation of horizontal PGAs of the 12 September 2016 *M*_*L*_ 5.8 earthquake, the largest earthquake in the Korean Peninsula since 1978. The PGAs at local and regional distances agree well with the theoretical curve, supporting the accuracy of the theoretical curve at short distances. (**c**) Spatial distribution of the horizontal PGAs. The locations of the stations are marked with triangles. The figure was created using GMT 4.5.14 (https://www.soest.hawaii.edu/gmt/) and Gnuplot 5.0 (https://www.gnuplot.info).

We collect the PGAs and PGVs from local and regional seismograms for the 6th UNE. We compare the observed ground motions (PGAs, PGVs) with the theoretical reference curves for an *m*_*b*_ 6.3 event (Fig. 3). The observed PGAs and PGVs agree with the theoretical variations. We additionally fit the observed ground motions with a theoretical curve that is found to have a larger magnitude by 0.2 from horizontal PGAs and 0.3 from vertical PGAs. The observation suggests that the theoretical curves represent the ground motions reasonably (see Supplementary Materials). The theoretical curve is compared with the observed ground motions of the 12 September 2016 *M*_*L*_5.8 earthquake for verification at local and near-regional distances (Supplementary Materials Fig. 3). The observation suggests that the theoretical curves for ground motion (PGA and PGV) represent the observed ground motions reasonably at local and near-regional distances.

We infer the ground-motion levels around the UNE test site from the theoretical ground-motion curves. The horizontal and vertical PGAs induced by the 6th UNE are estimated to be 169.0 m/s^2^ (22.9–1247.8 m/s^2^ at a 95% confidence level) and 150.8 m/s^2^ (20.8–1091.7 m/s^2^ at a 95% confidence level) at a hypocentral distance of 1 km. The PGAs at a hypocentral distance of 10 km are 5.8 m/s^2^ (0.8–42.8 m/s^2^ at a 95% confidence level) in the horizontal components and 4.2 m/s^2^ (0.6–30.4 m/s^2^ at a 95% confidence level) in the vertical component. The inferred ground motion levels in the UNE test site appear to be stronger than those observed in the Japanese islands during the 2011 *M*_*W*_9.0 Tohoku-Oki megathrust earthquake. The peak horizontal PGA reached 27.7 m/s^2^ on the coast nearest to the megathrust earthquake^23^.

The levels of ground motions by an explosion can be inferred using a scaled yield-acceleration equation^24^ (see, Methods). A recent study suggests the yield of the 6th UNE to be 230 kt^25^. According to a *m*_*b*_-yield relationship^11^, the yield of the 6th UNE is 109 kt. The ground acceleration for an explosion with yield of 230 kt is estimated to be 109 m/s^2^ (46–262 m/s^2^ at a 95% confidence level) at a hypocentral distance of 1 km. The ground acceleration for an explosion with yield of 109 kt is 79 m/s^2^ (33–189 m/s^2^ at a 95% confidence level). The levels of ground accelerations inferred from the scaled yield-acceleration equation are comparable to those inferred from the PGA attenuation equation. The observation suggests possible experience of strong ground motions around the test site by the 6th UNE.

We determine the dynamic stress changes from the PGVs. The dynamic stress changes at a distance of 1 km are 43.5 MPa (7.5–253.9 MPa at a 95% confidence level) in the horizontal component and 22.3 MPa (3.8–130.3 MPa) in the vertical component. The dynamic stress changes at a distance of 10 km are 1.5 MPa (0.3–8.6 MPa at a 95% confidence level) in the horizontal component and 0.8 MPa (0.1–4.9 MPa at a 95% confidence level) in the vertical component (see Supplementary Materials).

The levels of strong ground motions attenuate exponentially with distance (Fig. 3), with the level of ground motions at an epicentral distance of 2 km and 10 km being approximately half and 0.002 times, respectively, that in the epicenter. UNEs are typically detonated at shallow depths of approximately 1 km, while natural earthquakes occur at depths greater than several kilometers. Thus, ground motions by the UNEs may be much stronger than those by natural earthquakes of similar size. The 6th UNE produced unprecedented strong ground motions around the UNE test site.

**Figure 4:**
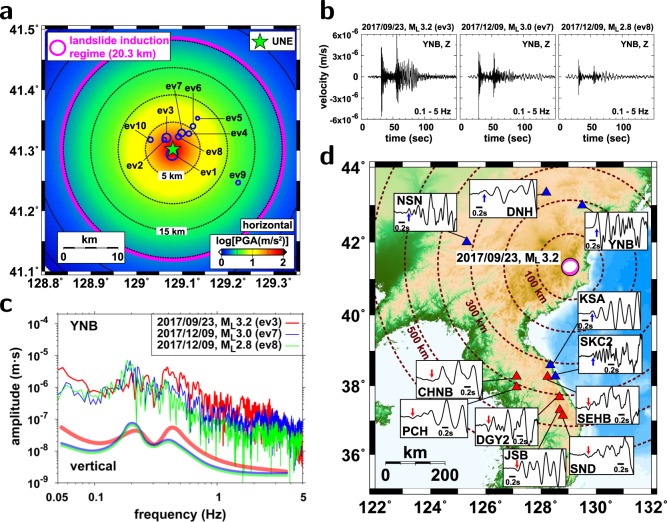
Post-UNE seismic events and their properties. (**a**) Spatial distribution of post-UNE events and horizontal peak ground accelerations. The seismic events are located in the landslide induction regime. (**b**) Vertical velocity seismic waveform records in 0.1–5 Hz for three post-UNE events (ev3, ev7, ev8) at station YNB. (**c**) Comparison of displacement spectra of three post-UNE events (ev3, ev7, ev8). The displacement spectra of ev7 and ev8 display peak energies at approximately 0.2 Hz. (**d**) *P* waveforms and polarities at regional stations. Stations at distances less than ~330 km present positive *P* polarities, and those at longer distances display negative *P* polarities. The figure was created using GMT 4.5.14 (https://www.soest.hawaii.edu/gmt/) and Adobe Illustrator CS6 (http://www.adobe.com/kr/products/illustrator.html).

Reports indicate that landslides may occur when the PGA is greater than 0.7 m/s^2^ and that there is the potential for serious landslides to occur when PGA > 2 m/s^2^ ^26^. The strong ground motions by the UNE have the potential to induce landslides and surface subsidence around the test site. The PGA levels by the 6th UNE are greater than 0.7 m/s^2^ up to distances of ~20.3 km (Fig. 4). On the other hand, the levels of ground motions by the 5th UNE are lower than than those by the 6th UNE, incorporating landslides around the UNE test site rarely (Supplementary Materials Fig. 3).

### Post-UNE seismic events

Severe subsidence at the detonation site followed the 6th UNE^27^. Ten events with magnitudes of greater than 2.0 occurred for 5 months since the 6th UNE (Fig. 1). The magnitudes of the events were *M*_*L*_2.5–4.1. A shallow seismic event of magnitude *M*_*L*_4.1 (*M*_*W*_4.5) occurred at a location ~700 m to the south of the 6th UNE 8 min 32 s after the 2017 UNE due to local post-explosion compaction^28^. A seismic event with magnitude of *M*_*L*_3.2 occurred in 20 days after the UNE (September 23, 2017). Successive seismic events followed in the region (Fig. 1).

The hypocentral parameters of the post-UNE events were refined by VELHYPO. The post-UNE events were clustered at a region to the north of the detonation site. The source locations are placed around the northern flanks of the mountain (Fig. 1). The post-UNE events presented characteristic shallow source depths less than 3 km below the surface (see Supplementary Materials). The source depths are shallower than the typical focal depths of natural earthquakes in the Korean Peninsula. The natural earthquakes in the Korean Peninsula typically nucleate at depths between 3 and 20 km, which is typical of a stable intraplate regime^3^.

A recent study based on a *P* polarity analysis of regional seismic records suggested the source mechanism of the 23 September 2017 *M*_*L*_3.2 event to be strike slip^10^. The seismic waveforms of an *M*_*L*_3.2 event are weak in regional distances, causing difficulty to determine the source mechanism stably from *P* polarities.

We conduct long-period waveform inversions for the 23 September 2017 *M*_*L*_3.2 post-UNE event (see Supplementary Materials). The long-period waveforms present relatively low signal-to-noise ratios in regional distances, causing low variance reduction. The full moment tensor solution is composed of 47% double-couple component, 46% CLVD component, and 7% isotropic component (see Supplementary Materials). The seismic moment is 3.79 × 10^14^ N · m. The moment magnitude was *M*_*W*_3.7. The deviatoric solution is close to the full moment tensor solution, displaying comparable strengths of double-couple and CLVD components. The synthetic waveforms for the full moment tensor solution fit to the observed waveforms better than those for the double couple solution (see Supplementary Materials). The full moment tensor solution may suggest a combined source with vertical mass compaction (collapse) and lateral mass expansion (outflow).

The spatial distribution of stations in the Korean Peninsula provides a unique dataset in local and near-regional distances. We observe distance-dependent *P*-polarity changes (Fig. 4). The *P* waves at distances of less than ~330 km display positive polarity, while those at farther distances present negative polarity. The observation agrees with the source mechanism inferred from the long-period waveform inversion.

We examine three clustered events (ev3, ev7, ev8) with magnitudes of *M*_*L*_3.2, 3.0, and 2.8. The 23 September 2017 *M*_*L*_3.2 event and other post-UNE events displayed strong *P* and relatively weak *S* wavetrains in local stations (Fig. 4). Dispersive *Rg* waves followed the *S* waves. Three nearby post-UNE events (ev3, ev7, ev8) presented similar waveforms.

The frequency contents of the events were similar, presenting prominent energy at frequencies of 0.05–0.5 Hz (Fig. 4). The 23 September 2017 event (ev3) displayed stronger energy in ~0.8 Hz than the other two events (ev7, ev8). On the other hand, the events following the *M*_*L*_3.2 event (ev7, ev8) showed spectral contents with peak energy at ~0.2 Hz (Supplementary Materials Fig. 4).

The events are placed at distances of less than ~15 km, mostly within 10 km from the 6th UNE (Fig. 4). The events occurred at a region with strong ground motions that may induce landslides on the surface. The shallow source depths and high CLVD-component composition suggest that the observed seismic events may not be typical natural earthquakes. The shallow source depths suggest that the medium was perturbed effectively by strong transient waves from the 6th UNE. The observed features suggest that the post-UNE events may be a result of high medium weakening by the 6th UNE.

## Discussion and Conclusions

The 6th North Korean UNE in 3 September 2017 was the largest detonation test conducted in North Korea to date. Strong ground motions were produced by the UNE. The horizontal peak ground accelerations on the detonation site were expected to be tens to hundreds m/s^2^ which were stronger than those on inland regions for the 2011 M9.0 Tohoku-Oki megathrust earthquake. The estimated levels of ground motions suggest that the medium of a wide region could be affected with cracking and collapse.

A series of shallow seismic events followed the 6th UNE to the north of the UNE test site. The source depths of the events were less than 3 km, which is shallower than the usual focal depths of natural earthquakes in the Korean Peninsula. The post-UNE events occurred in an area with a radius of ~15 km from the 6th UNE where the medium might be highly perturbed by the strong ground motions. The source mechanism of the 23 September 2017 *M*_*L*_3.2 post-UNE event suggests a combined source with comparable strengths of double-couple and CLVD components. The characteristic shallow source depths, nonpure double-couple source mechanism, and event clustering in near distances from the detonation site suggest that the post-UNE events might be produced by the release of tectonic-loading stress in the weakened medium with possible medium collapses and mass displacements.

Noble gases, radionuclides, and ionospheric disturbances were detected to a limited extent in earlier North Korean UNE tests^29,30^, which suggests that the radioactive matter from the UNEs might be well contained in the tunnels. The strong ground motions around the UNE test site might affect the medium significantly. Additionally, the strong transient waves from the 6th UNE could affect the magma chamber of Mount Baekdu, which is located 115 km away from the detonation site.

## Methods

We refined the hypocentral parameters of the UNEs and seismic events using VELHYPO^18,19^. VELHYPO combines a conventional hypocentral-parameter inversion method with a velocity-model refinement scheme. The method is based on *P* and *S* arrival times. The method searches for an optimum 1-D velocity model yielding minimum misfit errors in hypocentral-parameter inversion. The number, thicknesses, and velocities of layers can be defined. The method is useful for inversion in regions where velocity structures are not well known^13,14,19^. We implement a reported 1-D velocity model as an initial model^31^ and update the velocity structure in the hypocentral-parameter inversion.

The focal mechanism solutions of the UNEs and seismic events are determined using a long-period waveform inversion^13,32,33^. The bandpass filter range for the long-period waveform inversion is set to 0.04–0.07 Hz with slight adjustment considering the frequency contents of the waveforms.

The peak ground acceleration (PGA) and peak ground velocity (PGV) attenuate with distance. The ground-motion amplitudes (PGA and PGV) satisfy the relationship^1,34,35^.

1 $$\mathrm{log}\,{G}_{i,j,k,l}={A}_{i,j,l}+{B}_{i,j}\,\mathrm{log}\,{r}_{k,l}+{C}_{i,j}{r}_{k,l},$$

where *G*_*i*,*j*,*k*,*l*_ (*i* = PGA, PGV, *j* = *h*, *v*) is the peak ground motion (PGA or PGV) in the horizontal or vertical component at station *k* for event *l* at the hypocentral distance of *r*_*k*,*l*_, *A*_*i*,*j*,*l*_ is a constant calibrated for event size, *B*_*i*,*j*_ is a constant for geometrical spreading, and *C*_*i*,*j*_ is a constant for anelastic absorption. The PGA is in m/s^2^, the PGV is in m/s, and the distance *r* is in km (see Supplementary Materials). The constants are presented in the Supplementary Materials.

The ground motions by a underground explosion in hard rocks can be inferred using a scaled yield-acceleration equation^24^

2 $$A=9.29\times {10}^{6}\times {Y}^{-1/3}\times {(r\times {Y}^{-1/3})}^{-2.32},$$

where *A* is the acceleration in gravity acceleration (*g*), *r* is the distance in meters, and *Y* is the yield in kt. The yields of North Korean UNEs can be inferred from a magnitude-yield relationship^11^

3 $${m}_{b}=1.0125\,\mathrm{log}(Y)-0.7875\,\mathrm{log}(h)+5.887,$$

where *m*_*b*_ is the body wave magnitude, and *h* is the detonation depth in m. We set the depth to be 760 m for the 6th UNE considering the elevation difference between the tunnel entrance and surface^10^.

The peak dynamic stress change *σ*_*dyn*_ induced by ground motions can be calculated by^1,36,37^.

4 $${\sigma }_{dyn}=\mu \frac{{\dot{u}}_{peak}}{\beta },$$

where *μ* is the shear modulus, ${\dot{u}}_{peak}$ is the peak ground velocity, and *β* is the shear wave velocity. We set the shear modulus (*μ*) to 34.95 GPa, the shear velocity (*β*) to 3.58 km/s, and the density to 2750 kg/m^3^, considering the upper crustal properties of the Korean Peninsula.

The energy exerted by a seismic source is estimated using the moment magnitude, *M*_*W*_:

5 $$E={10}^{1.5\cdot ({M}_{W}+3.2)},$$

where *E* is the energy exerted by the source in Joules (=N · m).

## Supplementary information

supplementary materials
