# Supplementary material for: Seismic detection of strong ground motions by MW5.6 North Korean nuclear explosion
Source: Sci Rep. 2019 Mar 26;9:5124. doi: 10.1038/s41598-019-41627-x (PMC6435681; doi:10.1038/s41598-019-41627-x)
Supplement: Supplementary file 1 — supplementary materials [file 41598_2019_41627_MOESM1_ESM.pdf]

Supplementary Materials for

Seismic detection of strong ground motions by M_W5.6 North Korean nuclear explosion

Tae-Kyung Hong[a,*], Junhyung Lee[a], Seongjun Park[a], Hyun Ho Yoon[a], Woohan Kim[b], and Jin Soo Shin[c]

Affiliation and address:

[a]Yonsei University, Department of Earth System Sciences, 50 Yonsei-ro, Seodaemun-gu Seoul 120-749, South Korea.

[b]Gyeongsang National University, Department of Earth and Environmental Sciences and RINS, Jinju, Gyeongsangnam-do 660-701, South Korea.

[c]Earthquake Research Center, Korea Institute of Geoscience and Mineral Resources, 92 Gwahang-no, Yuseong-gu, Daejeon 305-350, South Korea.

Correspondence to:

tkhong@yonsei.ac.kr (Tae-Kyung Hong)

North Korean nuclear explosion tests

North Korea conducted 6 underground nuclear explosion (UNE) tests since 2000. The UNE tests were made in 9 October 2006 (1st), 25 May 2009 (2nd), 12 February 2013 (3rd), 6 January 2016 (4th), 9 September 2016 (5th), and 3 September 2017 (6th) (Fig. S1). The seismic magnitudes of the UNEs were m_b 4.3, 4.7, 5.1, 5.1, 5.3, and 6.3 (Hong and Rhie, 2009; Shin et al., 2010; Zhao et al., 2012; Zhang and Wen, 2013; Liu et al., 2018; Tian et al., 2018). The isotropic moment of the first UNE in 2006 was 3.1×10^{14} N·m (Koper et al., 2008). The total moment of the second UNE in 2009 was 6.3×10^{15} N·m (M_W4.5) (Ford et al., 2009). The Lg body-wave magnitudes of the five UNEs before the 6th UNE were $m_b(Lg)$ 3.9, 4.5, 4.9, 4.7, and 4.8 (Zhao et al., 2016, 2017). The surface-wave magnitudes of the five UNEs before the 6th UNE were M_S 2.92, 3.65, 3.94, 4.05, and 4.23 (Zhao et al., 2017).

Analysis

The peak ground acceleration (PGA) and peak ground velocity (PGV) attenuation satisfy an equation (Atkinson and Boore, 1995; Marin et al., 2004; Hong et al., 2016):

$$\log G_{i,j,k,l} = A_{i,j,l} + B_{i,j} \log r_{k,l} + C_{i,j}\, r_{k,l}, \tag{1}$$

where $G_{i,j,k,l}$ ($i =$PGA, PGV, $j = h, v$) is the peak ground motion (PGA or PGV) in the horizontal or vertical component at station k for event l at the hypocentral distance of $r_{k,l}$, $A_{i,j,l}$ is a constant calibrated for event size, $B_{i,j}$ is a constant for geometrical spreading, and $C_{i,j}$ is a constant for anelastic absorption. The PGA is in m/s^2, the PGV is in m/s, and the distance r is in km.

The constants for the ground motion attenuations were determined using the seismic records for earthquakes in the Korean Peninsula (Hong et al., 2016). The constants for PGA attenuation curves are found to be $B_{PGA,h} = -1.44$, $B_{PGA,v} = -1.54$, $C_{PGA,h} = -0.00211$, and $C_{PGA,v} = -0.00164$, and those for PGV attenuation curves are $B_{PGV,h} = -1.46$, $B_{PGV,v} = -1.52$, $C_{PGV,h} = -0.000939$, and $C_{PGV,v} = -0.000739$. The peak ground motions agree well with the regression curves (Fig. S2). The residuals between the observed PGAs and regression curves are clustered around zero. The source-strength constants satisfy (Hong et

al., 2016)

$$A_{PGA,h} = -0.318 + 0.394m_b, \quad A_{PGA,v} = -0.273 + 0.372m_b,$$
$$A_{PGV,h} = -3.388 + 0.620m_b, \quad A_{PGV,v} = -3.631 + 0.605m_b. \tag{2}$$

The spatial distribution of ground motions induced by the 2017 UNE is presented in Fig. S3. The PGVs in a hypocentral distance of 1 km are estimated to be 4.46 m/s (0.76-26.01 m/s at a 95 % confidence level) in horizonal components and 2.29 m/s (0.39-13.35 m/s at a 95 % confidence level) in vertical component (Fig. S2). The PGVs in a hypocentral distance of 10 km are estimated to be 0.15 m/s (0.03-0.89 m/s at a 95 % confidence level) in horizonal components and 0.09 m/s (0.02-0.50 m/s at a 95 % confidence level) in vertical component. The PGVs in a hypocentral distance of 100 km are estimated to be 0.004 m/s (0.0007-0.025 m/s at a 95 % confidence level) in horizonal components and 0.003 m/s (0.0005-0.016 m/s at a 95 % confidence level) in vertical component.

The PGAs in a hypocentral distance of 1 km are estimated to be 169.0 m/s^2 (22.9-1247.8 m/s^2 at a 95 % confidence level) in horizonal components and 150.8 m/s^2 (20.8-1091.7 m/s^2 at a 95 % confidence level) in vertical component. The PGAs in a hypocentral distance of 10 km are estimated to be 5.9 m/s^2 (0.8-43.4 m/s^2 at a 95 % confidence level) in horizonal components and 4.2 m/s^2 (0.6-30.4 m/s^2 at a 95 % confidence level) in vertical component. The PGAs in a hypocentral distance of 100 km are estimated to be 0.14 m/s^2 (0.02-1.02 m/s^2 at a 95 % confidence level) in horizonal components and 0.09 m/s^2 (0.01-0.63 m/s^2 at a 95 % confidence level) in vertical component.

The dynamic stress changes in a distance of 1 km are 43.5 MPa (7.5-253.9 MPa at a 95 % confidence level) in horizontal component, and 22.3 MPa (3.8-130.3 MPa at a 95 % confidence level) in vertical component. The dynamic stress changes in a distance of 10 km are 1479.7 kPa (253.6-8635.1 kPa at a 95 % confidence level) in horizontal component, and 835.9 kPa (143.2-4878.3 kPa at a 95 % confidence level) in vertical component. The dynamic stress changes in a distance of 100 km are 42.2 kPa (7.2-246.5 kPa at a 95 % confidence level) in horizontal component, and 27.3 kPa (4.7-159.1 kPa at a 95 % confidence level) in vertical component.

The theoretical ground-motion attenuation curve is compared with the observed ground

motions of the 12 September 2016 M_L5.8 earthquake in the Korean Peninsula, verifying the validity of the theoretical curve for representation of ground motions in short distances (Fig. S4).

The post-UNE events were relocated using VELHYPO (Fig. S5). The post-UNE events occurred around the flanks of mountains at shallow depths less than 2.5 km (Fig. S6). The 95 % confidence ranges of the estimated depth errors vary up to ±1327 m at a 95 % confidence level (Fig. S6). The events displayed peak seismic energy around 0.2 Hz (Fig. S7).

We performed long-period waveform inversions of the 23 September 2017 M_L3.2 post-UNE event. We determined a full moment tensor solution, deviatoric source solution, and double couple solution (Fig. S8). The full moment tensor solution and deviatoric solution are similar, presenting combined sources with substantial strengths of double-couple and compensated linear vector dipole (CLVD) components (Fig. S8). The full moment tensor solution is highly different from the double couple solution. Synthetic waveforms for the full moment tensor solution fit better than those for the double couple solution. The observation suggests that the 23 September 2017 M_L3.2 post-UNE event may not be a pure natural earthquake. The post-UNE events might occur in weakened medium with possible unidirectional mass displacements (Fig. S9).

References

Atkinson, M., and D. M. Boore (1995), Ground-motion relations for eastern North America, *Bulletin of the Seismological Society of America*, *85*, 17-30.

Chaves, E. J., T. Lay, and D.P. Voytan (2018), Yield estimate (230 kt) for a Mueller-Murphy model of the 3 September 2017, North Korean nuclear test (mbNEIC = 6.3) from teleseismic broadband P waves assuming extensive near-source damage, *Geophysical Research Letters*, *45*, 10,314-10,322.

Ford, S. R., D. S. Dreger, and W. R. Walter (2009), Source analysis of the Memorial Day explosion, Kimchaek, North Korea, *Geophysical Research Letters*, *36*, L21304, doi:10.1029/2009GL040003.

Hong, T.-K., E. Choi, S. Park, and J. S. Shin (2016), Prediction of ground motion and

dynamic stress change in Baekdusan (Changbaishan) volcano caused by a North Korean nuclear explosion, *Scientific Reports*, *6*, 21477, doi:10.1038/srep21477.

Hong, T.-K., and J. Rhie (2009), Regional source scaling of the 9 October 2006 underground nuclear explosion in North Korea, *Bulletin of the Seismological Society of America*, *99* (4), 2523-2540.

Koper, K. D., R. B. Herrmann, and H. M. Benz (2008), Overview of open seismic data from the North Korean event of 9 October 2006, *Seismological Research Letters*, *79*, 178-185.

Liu, J., L. Li, J. Zahradnik, E. Sokos, C. Liu, and X. Tian (2018), North Korea's 2017 test and its nontectonic aftershock, *Geophysical Research Letters*, *45*, 3017-3025.

Marin, S., J.-P. Avouac, M. Nicolas, and A. Schlupp (2004), A probabilistic approach to seismic hazard in metropolitan France, *Bulletin of the Seismological Society of America*, *94*, 2137-2163.

Perret, W. R., and R. C. Bass (1975), *Free-field ground motion induced by underground explosions*, Sandia Laboratories Report, SAND74-0252, Sandia National Laboratory, Albuquerque, New Mexico.

Shin, J. S., D.-H. Sheen, and G. Kim (2010), Regional observations of the second North Korean nuclear test on 2009 May 25, *Geophysical Journal International*, *180*, 243-250.

Tian, D., J. Yao, and L. Wen (2018), Collapse and earthquake swarm after North Korea's 3 September 2017 nuclear test, *Geophysical Research Letters*, *45*, 3976-3983.

Zhang, M., and L. Wen (2013), High-precision location and yield of North Korea's 2013 nuclear test, *Geophysical Research Letters*, *40*, 2941-2946.

Zhao, L.-F., X.-B. Xie, W.-M. Wang, and Z.-X. Yao (2012), Yield estimation of the 25 May 2009 North Korean nuclear explosion, *Bulletin of the Seismological Society of America*, *102*, 467-478.

Zhao, L.-F., X.-B. Xie, W.-M. Wang, J.-L. Hao, and Z.-X. Yao (2016), Seismological investigation of the 2016 January 6 North Korean underground nuclear test, *Geophysical Journal International*, *206*, 1487-1491.

Zhao, L.-F., X.-B. Xie, W.-M. Wang, N. Fan, X. Zhao, and Z.-X. Yao (2017), The 9 September 2016 North Korean Underground Nuclear Test, *Bulletin of the Seismological Society of America*, *107* (6), 3044-3051.

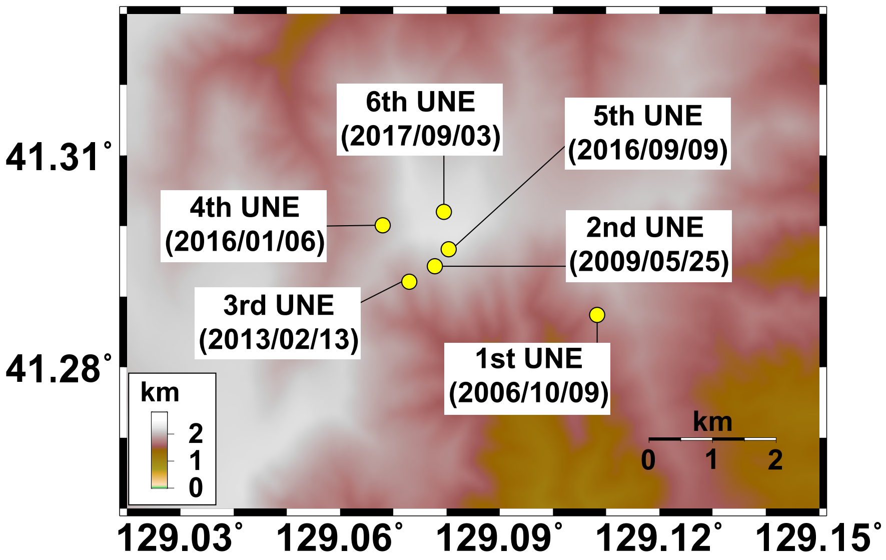

Figure S1. Map of six North Korean underground nuclear explosion tests since 2000. The surface topography is presented in color. The figure was created using GMT 4.5.14 (https://www.soest.hawaii.edu/gmt/) and Adobe Illustrator CS6 (http://www.adobe.com/kr/products/illustrator.html).

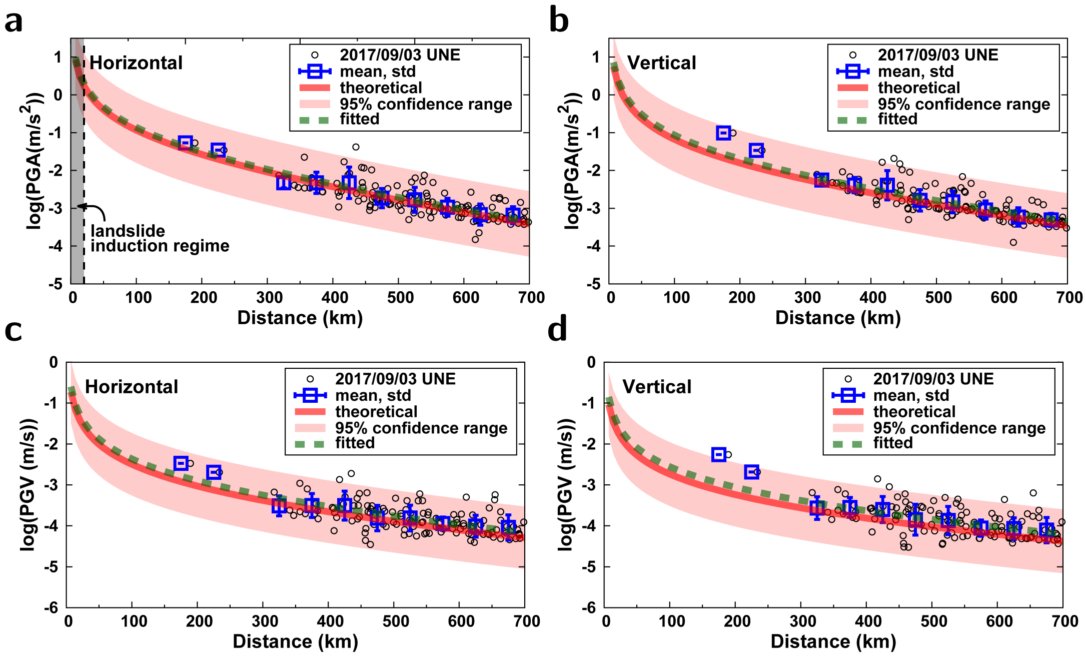

Figure S2. (a) Horizontal and (b) vertical peak ground accelerations (PGAs) and (c) horizontal and (d) vertical peak ground velocities (PGVs) observed in regional seismic stations for the 6th North Korean underground nuclear explosion in 3 September 2017. The observed PGAs and PGVs agree well with the theoretical curves. The figure was created using Gnuplot 5.0 (https://www.gnuplot.info).

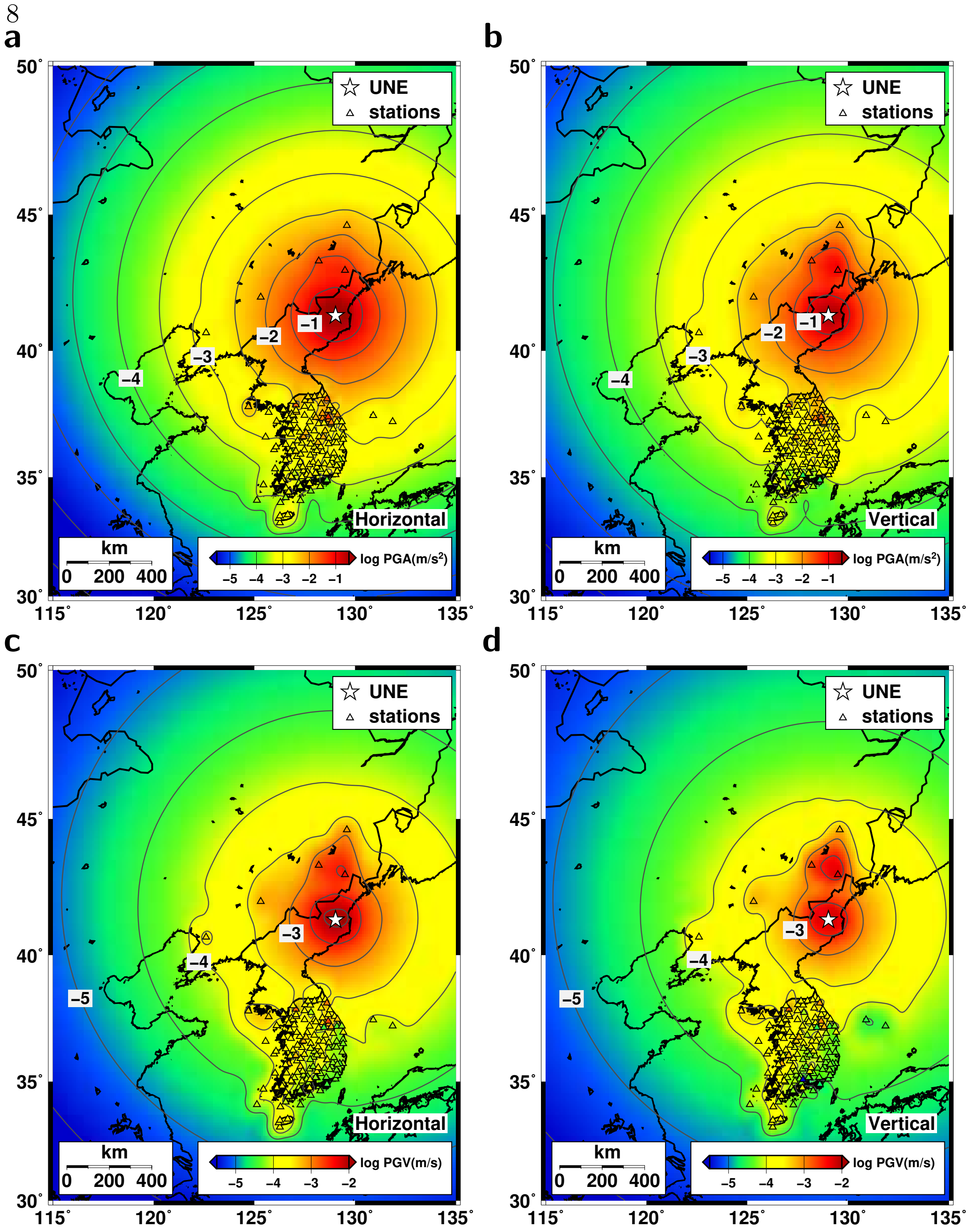

Figure S3. Spatial distribution of (a) horizontal and (b) vertical peak ground accelerations (PGAs) and (c) horizontal and (d) vertical peak ground velocities (PGVs). The locations of stations are marked with triangles. The figure was created using GMT 4.5.14 (https://www.soest.hawaii.edu/gmt/) and Adobe Illustrator CS6 (http://www.adobe.com/kr/products/illustrator.html).

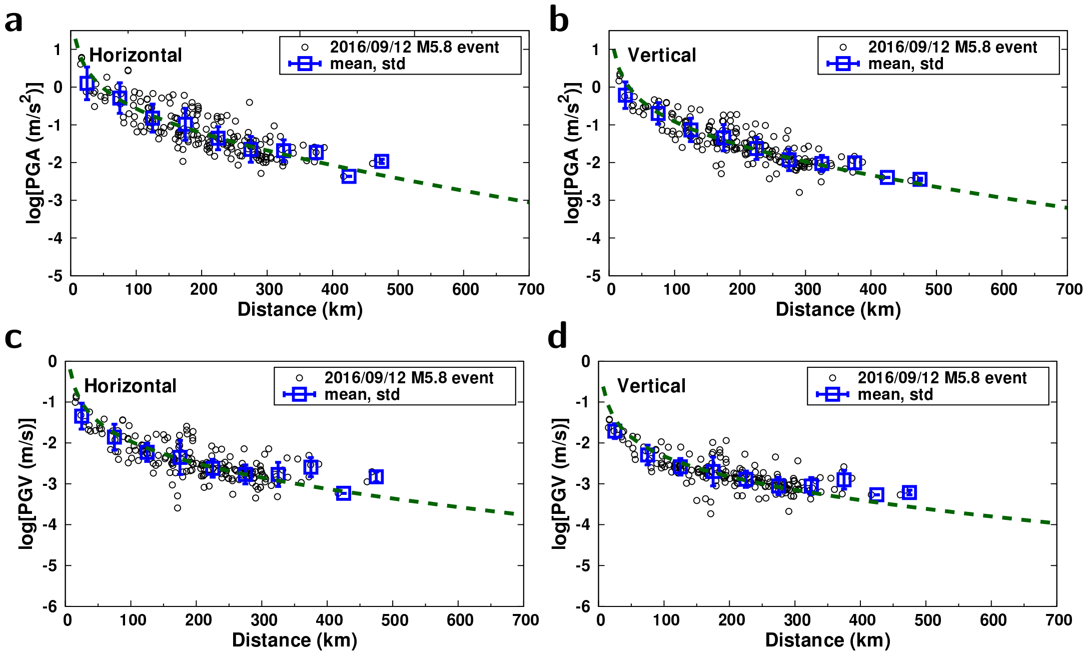

Figure S4. (a) Horizontal and (b) vertical peak ground accelerations (PGAs) and (c) horizontal and (d) vertical peak ground velocities (PGVs) observed in regional seismic stations for the 12 September 2016 M_L5.8 earthquake. The observed PGAs and PGVs agree well with the theoretical curves, suggesting the validity of the theoretical curves in short distances. The figure was created using Gnuplot 5.0 (https://www.gnuplot.info).

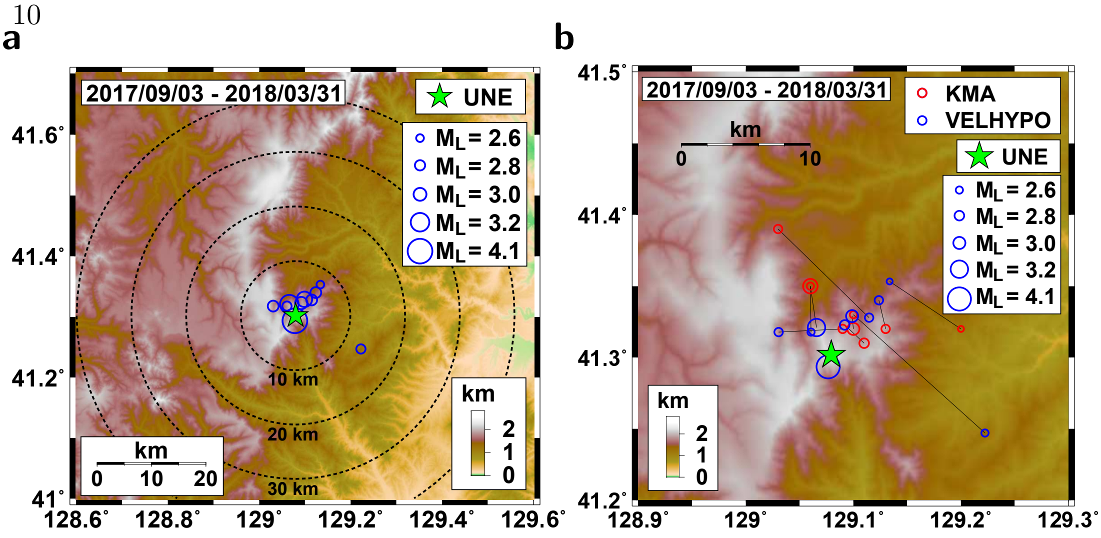

Figure S5. (a) Map of the 6th North Korean underground nuclear explosion (UNE) and post-explosion events (circles). (b) Comparison between refined and reported locations of post-explosion events. The reported locations were determined by Korea Meteorological Administration (KMA). The event locations were refined using VELHYPO. The figure was created using GMT 4.5.14 (https://www.soest.hawaii.edu/gmt/) and Adobe Illustrator CS6 (http://www.adobe.com/kr/products/illustrator.html).

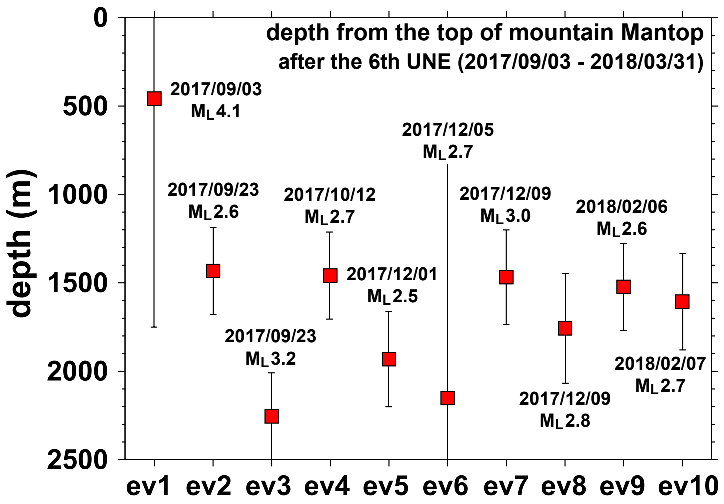

Figure S6. Vertical source locations of the post-UNE seismic events. The source depths are close to the surface. The figure was created using Gnuplot 5.0 (https://www.gnuplot.info).

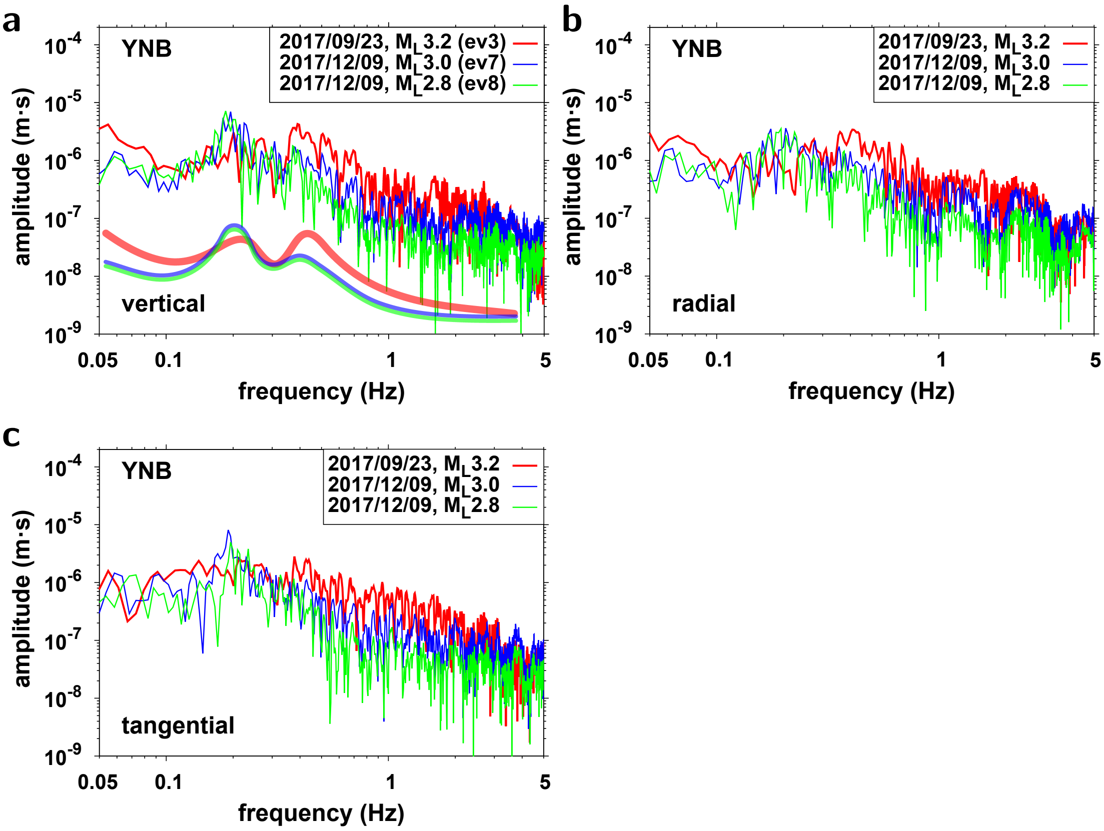

Figure S7. Comparison of displacement spectra between the 23 September 2017 M_L3.2 event and two other post-explosion events in (a) vertical, (b) radial, and (c) tangential components of station YNB. The figure was created using Gnuplot 5.0 (https://www.gnuplot.info).

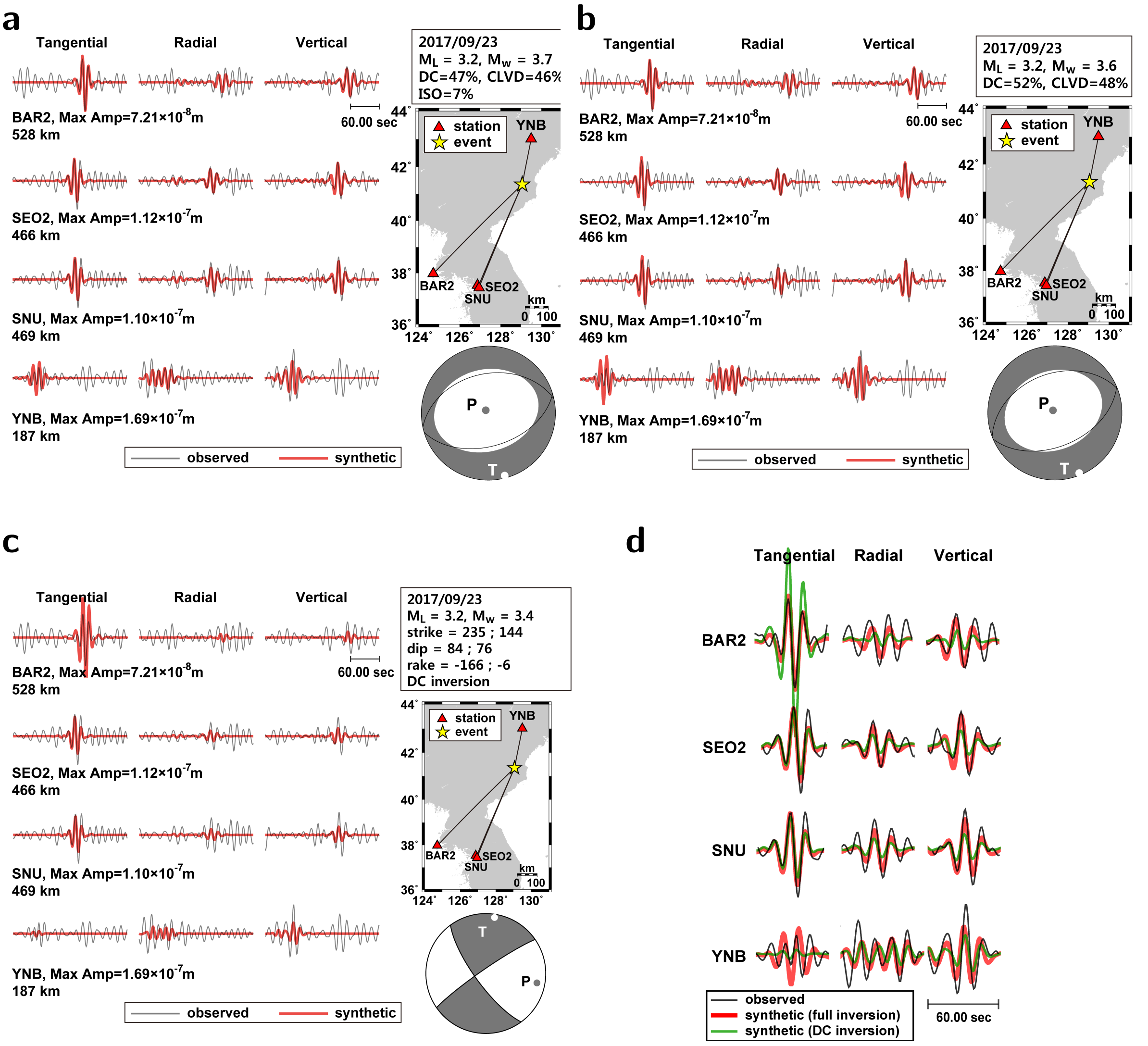

Figure S8. Long-period waveform inversions of the 23 September 2017 $M_L3.2$ post-explosion seismic event: (a) full moment tensor inversion, (b) deviatoric source inversion, (c) double-couple source inversion, and (d) comparison of inversion results. The full moment tensor solution and deviatoric solution present a combined source behavior with comparable strengths of double-couple and compensated linear vector dipole (CLVD) components. The synthetic waveforms for full moment tensor solution fit better than those for double couple solution. The figure was created using GMT 4.5.14 (https://www.soest.hawaii.edu/gmt/) and Adobe Illustrator CS6 (http://www.adobe.com/kr/products/illustrator.html).

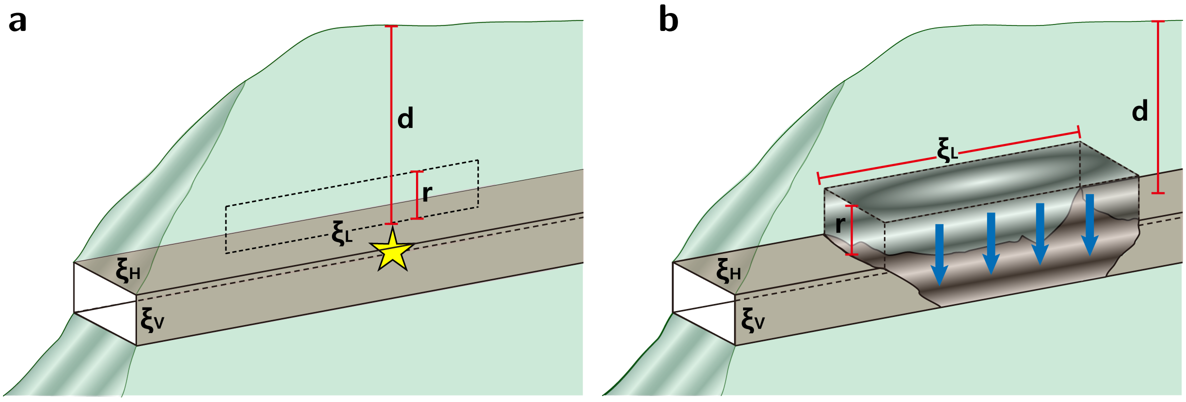

Figure S9. An example of shallow source with a considerable CLVD component. (a) A simplified tunnel model with the horizontal and vertical lengths of tunnel entrance to be ξ_H and ξ_V. The collapsed-tunnel length is ξ_L, the thickness of collapsed medium is r, and the depth of tunnel is d. (b) A schematic model of vertical tunnel collapse. The figure was created using Adobe Illustrator CS6 (http://www.adobe.com/kr/products/illustrator.html).
